# Long-Term Follow-Up Results of Mechanical Wrist Traction as Non-Invasive Treatment for Carpal Tunnel Syndrome

**DOI:** 10.3389/fneur.2021.668549

**Published:** 2021-09-06

**Authors:** Margreet Meems, Myrthe G. B. M. Boekhorst, Victor J. M. Pop

**Affiliations:** Department of Medical and Clinical Psychology, Centre of Research on Psychology in Somatic Diseases, Tilburg University, Tilburg, Netherlands

**Keywords:** Boston Carpal Tunnel Questionnaire, carpal tunnel syndrome, mechanical traction, carpal tunnel release surgery, non-invasive treatment

## Abstract

**Background:** For patients with carpal tunnel syndrome (CTS), the only long-term effective treatment option is carpal tunnel release surgery. Up to one-third report recurrent symptoms, and 12% needs repeated surgery. This study aimed to evaluate the long-term effects of mechanical traction as a non-invasive treatment option for CTS compared to care as usual.

**Methods:** Patients with electrodiagnostically confirmed CTS [*N* = 181; mean age, 58.1 (13.0) years; 67% women] were recruited from an outpatient neurology clinic in the Netherlands. Patients completed baseline questionnaires and randomized to the intervention group (12 treatments with mechanical traction, twice a week for 6 weeks) or care as usual. The primary clinical outcome measure was surgery during the 12-month follow-up. Secondly, we assessed symptom severity with the Boston Carpal Tunnel Questionnaire (BCTQ) at baseline and at the 12-month follow-up. Changes in CTS symptom severity between baseline and the 12-month follow-up were analyzed between groups using *t*-tests and a multiple linear regression analyses, adjusting for duration of complaints, age, gender, and symptom severity at baseline.

**Results:** At the 12-month follow-up, 35 of 94 (37%) patients in the intervention group had surgery, compared to 38 of 87 (44%) in the care-as-usual group ($\chi_{1}^{2}$ = 0.78, *p* = 0.377). Symptom severity and functional status scores did not significantly differ between the intervention (*n* = 81) and care-as-usual group (*n* = 55) at follow-up. For patients who did not have surgery, BCTQ scores decreased significantly more from baseline to the 12-month follow-up in the intervention group (*n* = 53) compared to patients in the care-as-usual group (*n* = 25). For patients who did not have surgery, belonging to the intervention group and a higher BCTQ score at baseline were related to a greater decrease in BCTQ scores from baseline to the 12-month follow-up, as well as symptom severity and functional status.

**Conclusions:** Mechanical traction is effective in reducing symptom severity compared to current conservative treatment options in standard care and can therefore benefit the large number of patients that prefer conservative treatment for CTS.

**Clinical Trial Registration:** Clinical Trials NL44692.008.13. Registered 19 September 2013, https://clinicaltrials.gov/ct2/show/NCT01949493

## Introduction

For patients with carpal tunnel syndrome (CTS), the only long-term effective treatment option is carpal tunnel release surgery (1). However, several disadvantages of receiving this surgery have been reported, such as surgery complications, suffering from sustained surgery-related pain, and weakness in the hands (2). In up to 30% of patients who underwent surgery, these symptoms can persist or recur, or patients may experience complications from surgery (3, 4). Up to 12% of patients require reoperation (3). Therefore, many patients postpone or opt out of surgery and turn to conservative treatment options. These treatment options are non-surgical and less invasive and can include exercise and mobilization interventions, oral non-steroidal drugs, corticosteroids (injections), and splinting (5–9). However, there is only short-term or limited evidence for the effectiveness of these interventions. Thus, there is a clear need for evidence of possible long-term benefits of an alternative, preferably non-invasive, therapy for CTS.

We have previously evaluated the short-term effect of mechanical traction as a non-invasive treatment option for CTS compared to care as usual in a randomized controlled trial (10). Results showed that patients receiving care as usual had an increased risk (2.3-fold risk) of having carpal tunnel release surgery compared to patients who were treated with mechanical traction at the 6-month follow-up. Furthermore, in both groups (care as usual and patients treated with mechanical traction), the symptom severity decreased significantly over time from baseline to the 6-month follow-up. The long-term effects, however, have not yet been reported.

The current study aimed to investigate the number of patients who had surgery at the 12-month follow-up in both the intervention and the care-as-usual groups. The secondary outcome was to evaluate the possible differences in CTS complaints between the intervention and care-as-usual groups. These differences were examined in all patients at follow-up as well as in a subgroup of patients who did not have surgery.

## Materials and Methods

### Participants

Patients with a diagnosis of CTS were recruited for the current study. The outpatient neurology clinic of VieCuri Medical Center in Venlo and Venray, the Netherlands, recruited patients from October 2013 to April 2015. We invited male and female patients between the ages of 18 and 80 who were diagnosed with CTS by means of electrodiagnostic testing to participate in the current study. Inclusion and exclusion criteria and invitation of eligible participants are described in detail elsewhere (11).

### Procedure

The research staff (MM) interviewed patients after they were included in the study, and patients completed paper-and-pencil questionnaires.

Next, 181 patients were included in the study and subsequently randomized into two groups: the mechanical traction intervention (*n* = 94) or care as usual (*n* = 87). The randomization procedure was previously described in detail elsewhere (11). The patients completed questionnaires at baseline and at the 3-, 6-, and 12-month follow-up. There were significantly more dropouts in the care-as-usual group (37%, *n* = 32) compared to the intervention group (14%, *n* = 13; $\chi_{1}^{2}$ = 12.7, *p* < 0.001, *V* = 0.27). There were no significant differences in baseline characteristics between patients who dropped out (*n* = 45) at 12 months and those who did not (*n* = 136).

#### Intervention: Phystrac Mechanical Traction Therapy

The intervention group received Phystrac mechanical traction therapy consisting of 12 treatment sessions (twice a week for a total of 6 weeks). Patients received treatment with the Phystrac mechanical traction device (type GR 10), which offers mechanical traction to the wrist using weights that range between 1 and 18 kg. Per affected hand, each session takes ~10–15 min to complete. The weight was set at 7 kg for men and 5 kg for women during the first session and increased with 2 kg for men and 1 kg for women for each consecutive session. This continued until a total of 13 kg for men or 10 kg for women was reached, or until the patient considered the mechanical traction to be uncomfortable. In general, 12 treatments with mechanical traction are considered sufficient for most patients. If CTS symptoms were not effectively reduced after 12 treatments with mechanical traction, patients could subsequently receive care as usual.

#### Control Group: “Care as Usual”

The control group received “care as usual.” Patients received standard treatment from their usual health care provider. They received treatment such as a wrist splint, local corticosteroid injections, or carpal tunnel release surgery. Patients and health care providers could also adopt an expectant approach. During the entire length of the study period, the types of treatment that patients from both groups obtained were documented using questionnaires and examination of their medical records.

### Outcome Measures

The primary outcome measure was whether patients had surgery at the 12-month follow-up, which was derived from the patients' medical records. Therefore, there were no missing data for this variable. The secondary outcome measure was long-term self-reported functional status and symptom severity in the two groups, which were measured at baseline and at the 12-month follow-up using the Boston Carpal Tunnel Questionnaire (BCTQ) (12, 13). Participants answered disease-specific items reflecting on a typical 24-h period in the past 2 weeks. The BCTQ has two subscales, namely, the Symptom Severity Scale (SSS) and the Functional Status Scale (FSS). The 11-item SSS assesses symptom severity. For the FSS, patients rate eight daily activities on their level of difficulty. The SSS and the FSS are rated on a 5-point scale. Both the SSS and FSS result in mean scores that range between 1 and 5, with higher scores reflecting greater impairment. The total BCTQ score is calculated as the mean of all items. The BCTQ is a suitable measure for CTS treatment outcome because it is responsive to clinically relevant change (12). The BCTQ is validated and is frequently used in studies with CTS symptom improvement over time (1), also in the Netherlands (14, 15).

A mean difference of 0.5 from before to after a possible intervention is regarded as the minimal clinical relevant difference that can be measured using the BCTQ (12). For the current study, a change of symptom severity between baseline and the 12-month follow-up was used as outcome variable.

### Statistical Analyses

Data are presented as mean ± standard deviation (SD) or percentages. Independent samples *t*-test and Chi^2^ tests were used to compare characteristics between groups. The effect sizes of significant differences between groups were evaluated using Cohen's d for *t*-tests and Cramer's V for Chi^2^ tests (16). For Cohen's *d*, 0.2, 0.5, and 0.8 were considered small, medium, and large effect sizes, respectively. For Cramer's V, 0.1, 0.3, and 0.5 were considered small, medium, and large effect sizes, respectively, for df = 1. After excluding patients who dropped out at the 12-month follow-up, changes in symptom severity and functional status were analyzed using BCTQ scores in the remaining sample (*n* = 136). Subgroup analyses were performed assessing changes in BCTQ scores in a subgroup of patients who did not receive surgery at the 12-month follow-up (*n* = 74). A multiple linear analysis was conducted to assess the impact of group (1 = intervention, 2 = care as usual) on the change in BCTQ scores from baseline to the 12-month follow-up. Age, gender (1 = male, 2 = female), duration of complaints (0 = 3 years or longer, 1 = shorter than 3 years), and BCTQ scores at baseline were added as covariates. The following assumptions of multiple regression analysis were checked: linear relationship, homoscedasticity, normality of distribution of residuals, and multicolinearity. Statistical analyses were performed using the Statistical Package of Social Science (SPSS, 25.0).

## Results

### Primary Outcome Measure: Effects of Mechanical Traction on Surgery During the 12-Month Follow-Up After Inclusion

At the 12-month follow-up, 35 of 94 (37%) patients in the intervention group had surgery, compared to 38 of 87 (44%) in the care-as-usual group ($\chi_{1}^{2}$ = 0.78, *p* = 0.377). Kaplan–Meier survival curves showed no significant group differences over time.

### Secondary Outcome Measure: Symptom Severity and Functional Status at the 12-Month Follow-Up

At the 12-month follow-up, symptom severity and functional status scores did not significantly differ between the intervention (*n* = 81) and care-as-usual groups (*n* = 55). BCTQ scores decreased in both the intervention and care-as-usual groups [−0.95 (0.78) and −0.89 (0.83)], respectively. There was no significant difference in change in BCTQ scores between the groups (*t* = −0.45, *p* = 0.650).

### Subgroup Analyses in Patients Who Did Not Receive Surgery

We subsequently compared change in symptom severity and functional status from baseline to the 12-month follow-up in patients of the intervention group who did not have surgery (*n* = 53) with the change in symptom scores of patients in the care-as-usual group who did not have surgery (*n* = 25).

The baseline characteristics of this subsample are shown in Table 1. No significant differences were found between the intervention and care-as-usual group. However, at the 90% significance level, patients in the care-as-usual group were more often male (χ^2^ = 3.25, *p* = 0.071, *V* = 0.20) while patients in the intervention group reported regular alcohol intake more often (χ^2^ = 3.05, *p* = 0.081, *V* = 0.20). Also, at baseline, patients in the care-as-usual group [2.53 (0.73)] had lower SSS scores at a 90% significance level compared to the intervention group [2.88 (0.84)] (*t* = 1.81, *p* = 0.07, *d* = 0.44). In the intervention group, two patients received a steroid injection during the 12-month follow-up, and two patients had physical therapy. In the care-as-usual group, two patients received a steroid injection. At the 12-month follow-up, 12 patients in the intervention group used a wrist splint regularly at night, compared to nine patients in the care-as-usual group.

**Table 1:** Baseline characteristics of the 78 participants who did not receive surgery at the 12-month follow-up.

	**Total (** ***n*** **= 78)**		**Intervention (** ***n*** **= 53)**		**Care as usual (** ***n*** **= 25)**		***p***	
	**Mean (SD)**	***n* (%)**	**Mean (SD)**	***n* (%)**	**Mean (SD)**	***n* (%)**	***t***	**χ^2^**
**Demographic features**								
Age (in years)	59.7 (11.0)		59.8 (11.9)		59.6 (9.2)		0.943	
Sex								0.071
Male		30 (38.5)		24 (45.3)		6 (24.0)		
Female		48 (61.5)		29 (54.7)		19 (76.0)		
Educational level								0.758
Low		64 (82.1)		43 (81.1)		21 (84.0)		
High		14 (17.9)		10 (18.9)		4 (16.0)		
Marital status								0.658
With partner		60 (76.9)		40 (75.5)		20 (80.0)		
**CTS related**								
Duration of complaints								0.939
<3 years		62 (79.5)		42 (79.2)		20 (80.0)		
>3 years		16 (20.5)		11 (20.8)		5 (20.0)		
Dominant hand involved								0.401
No		16 (20.5)		13 (24.5)		3 (12.0)		
Yes		19 (24.4)		13 (24.5)		6 (24.0)		
Both hands		43 (55.1)		27 (51.0)		16 (64.0)		
Direct relative with CTS		21 (26.9)		14 (26.4)		7 (28.0)		0.883
Paid hand labor								0.146
No		47 (60.3)		29 (54.7)		18 (72.0)		
Heavy		31 (39.7)		24 (45.3)		7 (18.0)		
SSS score	2.76 (0.82)		2.88 (0.84)		2.52 (0.73)		0.074	
FSS score	2.40 (0.93)		2.44 (0.91)		2.32 (0.99)		0.571	
BCTQ score	2.61 (0.80)		2.69 (0.81)		2.43 (0.77)		0.173	
**Lifestyle habits**								
Smoking		13 (16.7)		9 (17.0)		4 (16.0)		0.914
Alcohol		19 (24.4)		16 (30.2)		3 (12.0)		0.081
BMI	29.6 (5.63)		29.7 (6.15)		29.4 (4.44)		0.782	

**Figure 1:**
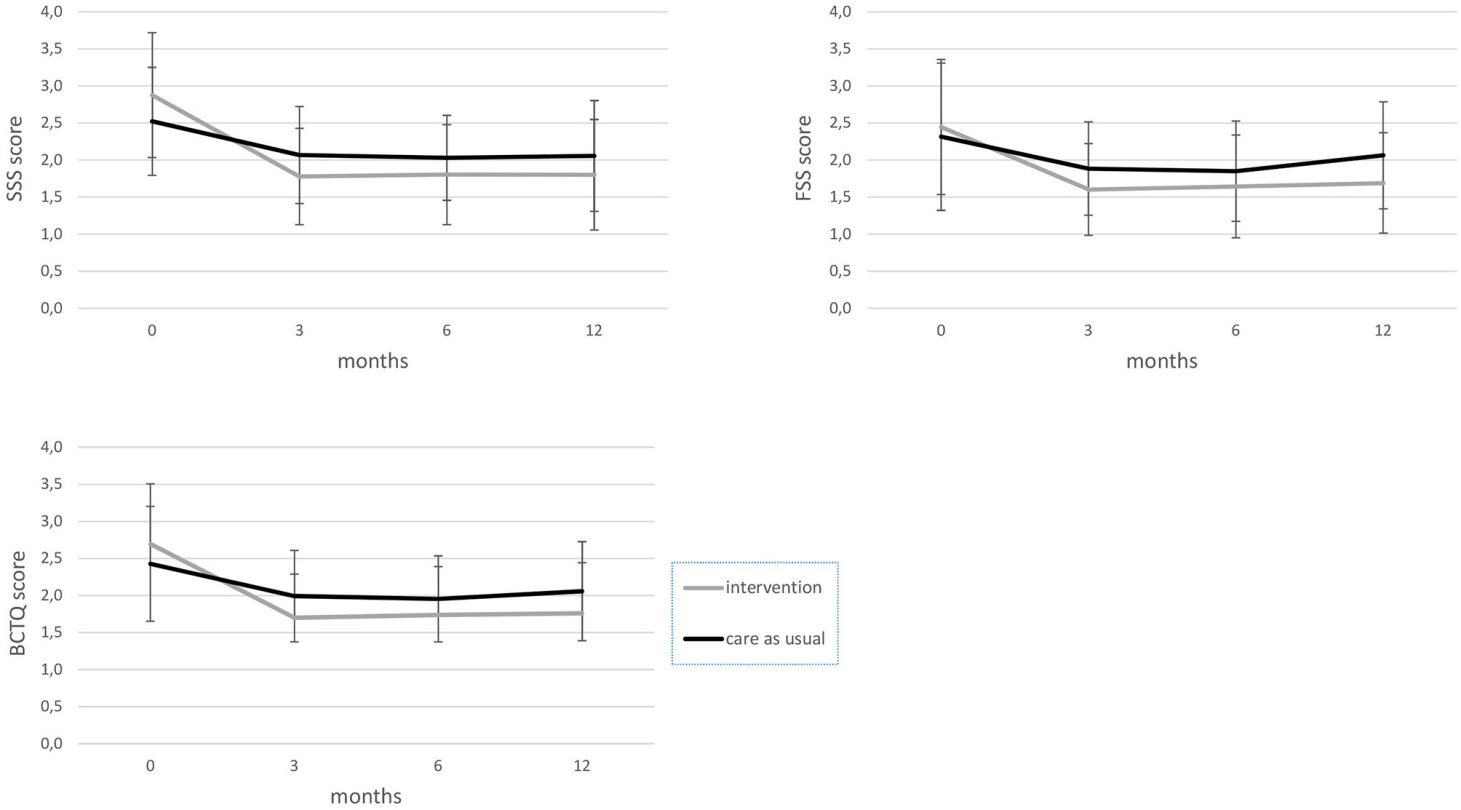
BCTQ, FSS, and SSS scores from baseline to the 12-month follow-up for the intervention and care-as-usual groups, excluding participants who had surgery. BCTQ, boston carpal tunnel questionnaire; FSS, functional status scale; SSS, symptom severity scale.

In these two subgroups of patients who did not receive surgery, BCTQ scores decreased significantly more in the intervention group compared to the care-as-usual group, as well as for SSS and FSS scores, with a medium–large effect size (see Figure 1; Table 2).

**Table 2:** Comparison of the difference in BCTQ scores between baseline and the 12-month follow-up between the intervention and care-as-usual groups, excluding participants who had surgery.

	**Difference score (SD) intervention group (*n* = 53)**	**Difference score (SD) care-as-usual group (*n* = 25)**	***t***	***p***	***d***
SSS	−1.07 (0.86)	−0.47 (0.70)	−3.08	0.003	0.77
FSS	−0.75 (0.87)	−0.25 (0.65)	−2.57	0.012	0.65
BCTQ	−0.94 (0.80)	−0.37 (0.59)	−3.17	0.002	0.81

### Linear Regression of Change in SSS, FSS, and BCTQ

To investigate the possible independent effect of belonging to the intervention group (yes/no) on the change in SSS, FSS, and BCTQ scores, three separate multiple linear regression analyses were performed with group, age, gender, duration of complaints, and symptom severity scores at baseline as predictor variables, and the change in SSS, FSS, and BCTQ scores as dependent variables. No violation of assumptions was found.

The first model explained 41.8% of the variance regarding the change in symptom severity from baseline to the 12-month follow-up [F_(5,72)_ = 10.65, *p* < 0.001]. Belonging to the intervention group and a higher SSS score at baseline were significantly and independently related to a greater decrease in SSS scores from baseline to the 12-month follow-up (see Table 3, model 1).

**Table 3:** Results of the multiple linear regression analysis predicting change in SSS (model 1), FSS (model 2), and BCTQ (model 3) scores.

	**Beta**	***t***	***p***
**Model 1**			
Group	0.213	2.243	0.028
Duration of complaints <3 years	−0.091	−0.990	0.326
Age	0.065	0.708	0.481
Gender	0.017	0.174	0.862
SSS at baseline	−0.580	−6.045	<0.001
**Model 2**			
Group	0.241	2.885	0.005
Duration of complaints <3 years	−0.062	−0.0758	0.451
Age	0.006	0.072	0.942
Gender	−0.010	−0.103	0.918
FSS at baseline	−0.670	−7.547	<0.001
**Model 3**			
Group	0.250	2.737	0.008
Duration of complaints <3 years	−0.079	−0.895	0.374
Age	0.045	0.501	0.618
Gender	0.000	0.004	0.997
BCTQ at baseline	−0.595	−6.307	<0.001

*Group (intervention = 1, care as usual = 2), duration of complaints (≥3 years = 0, <3 years = 1), and gender (male = 1, female = 2)*.

The second model explained 53.0% of the variance regarding the change in functional status from baseline to the 12-month follow-up [F_(5,72)_ = 16.25, *p* < 0.001]. Belonging to the intervention group and a higher FSS score at baseline were significantly and independently related to a greater decrease in FSS scores from baseline to the 12-month follow-up (see Table 3, model 2).

The third model explained 45.5% of the variance regarding the change in BCTQ scores from baseline to the 12-month follow-up [F_(5,72)_ = 12.01, *p* < 0.001]. Belonging to the intervention group and a higher BCTQ scores at baseline were significantly and independently related to a greater decrease in BCTQ scores from baseline to the 12-month follow-up (see Table 3, model 3).

## Discussion

The current study assessed the 12-month follow-up effects of mechanical traction for CTS patients compared to those receiving care as usual. Results showed that at the 12-month follow-up, there was no significant difference between the number of patients who had surgery in the intervention and care-as-usual group (37 and 44%, respectively). Moreover, symptom severity did not significantly differ between the intervention (*n* = 81) and care-as-usual group (*n* = 55) at follow-up. However, in the subsample of patients who did not have surgery at the 12-month follow-up, patients in the intervention group had a greater decrease in symptom severity from baseline to the 12-month follow-up compared to patients in the care-as-usual group. Lastly, belonging to the intervention group and symptom severity scores at baseline were significant predictors for the change in symptom severity scores within the group of patients who did not have surgery.

Evidence for the long-term effects (≥12 months) of conservative treatment on CTS symptom severity is scarce. A few studies have reported long-term results of splinting or steroid injections (17). Jarvik et al. (18) showed that the symptoms of surgery patients improved more than those who received conservative treatment (anti-inflammatory drugs, hand therapy, and ultrasound therapy). Both surgical and non-surgical groups improved over 12 months, but the decrease in CTS symptoms was significantly greater at the 12-month follow-up for patients who had surgery. However, the differences were small and of moderate clinical relevance. Another study by Fernández-de-Las Peñas et al. (19) compared surgery to physical therapy and showed that both groups had similar improvements in symptom severity at the 12-month follow-up. Both studies analyzed according to intention-to-treat, which makes it difficult to distinguish specific treatment effects.

In the current study, the change in symptoms severity (−1.07) and functional status (−0.75) in the intervention group is more than the minimal clinical relevant difference of 0.5 that can be measured using the BCTQ (12).

At the 12-month follow-up, 40% of all patients had surgery in the current study. Generally, more than half of patients with CTS try to avoid surgery (19). Most patients prefer non-invasive treatment options because of the recovery time after surgery and the chance of complications and recurrent symptoms. Patients and physicians can have several motives for choosing conservative treatment, such as patient's age, symptom severity, pregnancy, and the presence of comorbidities (20–23). The current study shows that mechanical traction is a conservative treatment option with promising long-term effectiveness.

Strengths and limitations of the current study have previously been discussed (11). One of the limitations is the lack of an objective indicator of improvement. The effectiveness of the interventions was not based on an objective measure such as electrodiagnostic testing but based on the patients' perception of symptoms. However, nerve conduction improves after intervention, but only moderately correlates to patient-reported symptom improvement (24). Therefore, electrodiagnostic testing is not sensitive enough to evaluate clinical change following intervention. Most studies use patient reported outcomes to evaluate treatment effect, such as the BCTQ, which is highly validated and sensitive to clinical change (12, 14, 18). Additionally, of the patients in the care-as-usual group who did not have surgery, 25 (51.0%) responded at the 12-month follow-up, compared to 53 (89.8%) in the intervention group. This could possibly lead to a response bias, where patients who do not have any symptoms fail to respond. We therefore compared baseline characteristics between the remaining patients in the intervention and control groups. Patients in the intervention group were more often male and reported a higher symptom severity at baseline at the 90% significance level. Moreover, symptom duration is associated with a negative outcome of conservative management (18). Therefore, these variables were adjusted for in the multiple linear regression analyses.

The intervention in the current study consisted of a 10- to 15-min session of mechanical traction per hand, twice a week during a period of 6 weeks. Future research should use a design with even longer follow-up, with special focus on symptom evaluation in the care-as-usual group to decrease the relatively high dropout. The mechanism for the effectiveness of mechanical traction treatment for CTS is still unknown. We expect that traction applied to the wrist reduces pressure in the carpal tunnel by improving blood microcirculation and reducing edema in the synovial tissue (9, 25). Future studies should focus in more detail (e.g., ultrasound) what the possible working mechanisms could be of mechanical traction. Also, it is a matter of speculation whether another intervention of 6 weeks after the 12-month follow-up in those with still higher symptom severity scores could even further improve treatment outcome. Mechanical traction is non-invasive, low in costs, and acceptable for CTS patients.

## Conclusions

For patients who do not opt for surgery, mechanical traction can be effective at reducing symptoms severity compared to current conservative treatment options in standard care. Mechanical traction can therefore benefit a large number of patients that prefer conservative treatment for CTS. Future studies should investigate the working mechanism and cost-effectiveness of (repeated) mechanical traction and identify possible patient subgroups that specifically benefit from mechanical traction.

## Data Availability Statement

The datasets presented in this article are not readily available due to privacy reasons, but are available from the corresponding author on reasonable request. Requests to access the datasets should be directed to m.g.b.m.boekhorst@uvt.nl.

## Ethics Statement

The studies involving human participants were reviewed and approved by the Medical Ethical Committee of the St. Elisabeth Hospital in Tilburg, the Netherlands in August 2013 (protocol #P1340). The patients/participants provided their written informed consent to participate in this study.

## Author Contributions

VP supervised the study. MM and VP were involved in the design of the study. MM worked on data acquisition and drafted the final manuscript. MM, MB, and VP were involved in analyzing and interpreting the data and critically revised the manuscript and approved the final version. All authors contributed to the article and approved the submitted version.

## Conflict of Interest

The authors declare that the research was conducted in the absence of any commercial or financial relationships that could be construed as a potential conflict of interest.

## Publisher's Note

All claims expressed in this article are solely those of the authors and do not necessarily represent those of their affiliated organizations, or those of the publisher, the editors and the reviewers. Any product that may be evaluated in this article, or claim that may be made by its manufacturer, is not guaranteed or endorsed by the publisher.

## References

[1] Gerritsen AA Uitdehaag BM van Geldere D Scholten RJ de Vet HC Bouter LM. Systematic review of randomized clinical trials of surgical treatment for carpal tunnel syndrome. Br J Surg. (2001) 88:1285–95. 10.1046/j.0007-1323.2001.01858.x 11578281

[2] Ashworth NL. Carpal tunnel syndrome. BMJ Clin Evid. (2010) 1114. PMC2907614 21718565

[3] Neuhaus V Christoforou D Cheriyan T Mudgal CS. Evaluation and treatment of failed carpal tunnel release. Orthop Clin North Am. (2012) 43:439–47. 10.1016/j.ocl.2012.07.013 23026459

[4] Uchiyama S Itsubo T Nakamura K Kato H Yasutomi T Momose T. Current concepts of carpal tunnel syndrome: pathophysiology, treatment, and evaluation. J Orthop Sci. (2010) 151:1–13. 10.1007/s00776-009-1416-x 20151245

[5] Huisstede BM Hoogvliet P Randsdorp MS Glerum S van Middelkoop M Koes BW. Carpal tunnel syndrome. Part I: effectiveness of nonsurgical treatments–a systematic review. Arch Phys Med Rehabil. (2010) 91:981–1004. 10.1016/j.apmr.2010.03.022 20599038

[6] Marshall S Tardif G Ashworth N. Local corticosteroid injection for carpal tunnel syndrome. Cochrane Database Syst Rev. (2007) 2:CD001554. 10.1002/14651858.CD001554.pub2 17443508 PMC12709571

[7] O'Connor D Marshall S Massy-Westropp N. Non-surgical treatment (other than steroid injection) for carpal tunnel syndrome. Cochrane Database Syst Rev. (2003) 1:CD003219. 10.1002/14651858.CD003219 12535461 PMC6486195

[8] Page MJ Massy-Westropp N O'Connor D Pitt V. Splinting for carpal tunnel syndrome. Cochrane Database Syst Rev. (2012) 7:CD010003. 10.1002/14651858.CD010003 22786532 PMC7389822

[9] Page MJ O'Connor D Pitt V Massy-Westropp N. Exercise and mobilisation interventions for carpal tunnel syndrome. Cochrane Database Syst Rev. (2012) 6:CD009899. 10.1002/14651858.CD009899 22696387 PMC11536321

[10] Meems M Spek V Kop WJ Meems BJ Visser LH Pop VJM. Mechanical wrist traction as a non-invasive treatment for carpal tunnel syndrome: a randomized controlled trial. Trials. (2017) 18:464. 10.1186/s13063-017-2208-9 29017511 PMC5634882

[11] Meems M Den Oudsten B Meems BJ Pop V. Effectiveness of mechanical traction as a non-surgical treatment for carpal tunnel syndrome compared to care as usual: study protocol for a randomized controlled trial. Trials. (2014) 15:180. 10.1186/1745-6215-15-180 24886455 PMC4039326

[12] Leite JC Jerosch-Herold C Song F. A systematic review of the psychometric properties of the boston carpal tunnel questionnaire. BMC Musculoskelet Disord. (2006) 7:78. 10.1186/1471-2474-7-78 17054773 PMC1624826

[13] Ortiz-Corredor F Calambas N Mendoza-Pulido C Galeano J Diaz-Ruiz J Delgado O. Factor analysis of carpal tunnel syndrome questionnaire in relation to nerve conduction studies. Clin Neurophysiol. (2011) 122:2067–70. 10.1016/j.clinph.2011.02.030 21454124

[14] Hoefnagels WA van Kleef JG Mastenbroek GG de Blok JA Breukelman AJ de Krom MC. Surgical treatment of carpal tunnel syndrome: endoscopic or classical (open)? A prospective randomized trial. Ned Tijdschr Geneeskd. (1997) 141:878–82. 9273452

[15] Peters-Veluthamaningal C Winters JC Groenier KH Meyboom-de Jong B. Randomised controlled trial of local corticosteroid injections for carpal tunnel syndrome in general practice. BMC Fam Pract. (2010) 11:54. 10.1186/1471-2296-11-54 20670438 PMC2921105

[16] Cohen J. A power primer. Psychologic Bull. (1992) 112:155–9. 10.1037/0033-2909.112.1.155 19565683

[17] Burton CL Chesterton LS Chen Y van der Windt DA. Clinical course and prognostic factors in conservatively managed carpal tunnel syndrome: a systematic review. Arch Phys Med Rehabil. (2016) 97:836–52. 10.1016/j.apmr.2015.09.013 26440776

[18] Jarvik JG Comstock BA Kliot M Turner JA Chan L Heagerty PJ. Surgery versus non-surgical therapy for carpal tunnel syndrome: a randomised parallel-group trial. Lancet. (2009) 374:1074–81. 10.1016/S0140-6736(09)61517-8 19782873

[19] Fernández-de-Las Peñas C Ortega-Santiago R de la Llave-Rincón AI Martínez-Perez A Fahandezh-Saddi Díaz H Martínez-Martín J. Manual physical therapy versus surgery for carpal tunnel syndrome: a randomized parallel-group trial. J Pain. (2015) 16:1087–94. 10.1016/j.jpain.2015.07.012 26281946

[20] Bessette L Keller RB Liang MH Simmons BP Fossel AH Katz JN. Patients' preferences and their relationship with satisfaction following carpal tunnel release. J Hand Surg Am. (1997) 22:613–20. 10.1016/S0363-5023(97)80117-7 9260615

[21] Jerosch-Herold C Mason R Chojnowski AJ. A qualitative study of the experiences and expectations of surgery in patients with carpal tunnel syndrome. J Hand Therap. (2008) 21:54–61. 10.1197/j.jht.2007.09.001 18215752

[22] Shifflett GD Dy CJ Daluiski A. Carpal tunnel surgery: patient preferences and predictors for satisfaction. Patient Prefer Adherence. (2012) 6:685–9. 10.2147/PPA.S36088 23055702 PMC3468169

[23] Meems M Truijens S Spek V Visser LH Pop V. Prevalence, course and determinants of carpal tunnel syndrome symptoms during pregnancy: a prospective study. BJOG. (2015) 122:1112–8. 10.1111/1471-0528.13360 25778497

[24] Schrijver HM Gerritsen AA Strijers RL Uitdehaag BM Scholten RJ de Vet HC. Correlating nerve conduction studies and clinical outcome measures on carpal tunnel syndrome: lessons from a randomized controlled trial. J Clin Neurophysiol. (2005) 22:216–21. 15933495

[25] Brunarski DJ Kleinberg BA Wilkins KR. Intermittent axial wrist traction as a conservative treatment for carpal tunnel syndrome: a case series. J Can Chiropr Assoc. (2004) 48:211–6. 17549120 PMC1769449

